# Health care policy that relies on poor measurement is ineffective: Lessons from the hospital readmissions reduction program

**DOI:** 10.1111/1475-6773.14161

**Published:** 2023-04-17

**Authors:** Ann M. Sheehy, Charles F. S. Locke, Nicole Bonk, Ronald L. Hirsch, W. Ryan Powell

**Affiliations:** ^1^ Division of Hospital Medicine, Department of Medicine Center for Health Disparities Research, University of Wisconsin School of Medicine and Public Health Madison Wisconsin USA; ^2^ Department of Medicine Johns Hopkins University School of Medicine Baltimore Maryland USA; ^3^ Division of Hospital Medicine, Department of Medicine University of Wisconsin School of Medicine and Public Health Madison Wisconsin USA; ^4^ R1 RCM, Inc. Murray Utah USA; ^5^ Division of Geriatrics and Gerontology, Department of Medicine Center for Health Disparities Research, University of Wisconsin School of Medicine and Public Health Madison Wisconsin USA

In 2009, Jencks and colleagues published their landmark New England Journal of Medicine paper on readmissions in the Medicare fee‐for‐service program. Their conclusion was simple: “Rehospitalizations among Medicare beneficiaries are prevalent and costly.”^1^ The next year, the Patient Protection and Affordable Care Act (ACA) was passed, including the Hospital Readmissions Reduction Program (HRRP) to improve healthcare quality by reducing hospital readmissions. As directed by law, since October 1, 2012, the U.S. Department of Health and Human Services Secretary has reduced payments to hospitals with excess 30‐day readmissions following index inpatient stays for common target conditions.^2^

The seemly logical origin of the HRRP followed the teaching of Peter Drucker who stated, “If you can't measure it, you can't improve it”—a quote featured prominently on the Centers for Medicare & Medicaid Services (CMS) Measures Management website.^3^ But like any initiative intended to improve quality, evaluation, and iteration is key to assessing and improving delivery.^4^ More than a decade later, the question now must be flipped to ask “We have measured it, have we improved it?” Unfortunately, due to flaws in the measure itself, lessons learned about readmission drivers, and the rapidly changing landscape of healthcare in the United States, readmissions have not improved as hoped. A simple PubMed search for “Hospital Readmissions Reduction Program” since 2012 yields more than 1200 citations—a body of literature representing a decade of trying—that fail to demonstrate consistent measurable improvements in hospital ability to meaningfully impact readmissions.^5^ In this commentary, we argue that the HRRP is in need of reform, and efforts to improve the HRRP must address measure shortcomings, including a new approach to support populations adversely impacted by social determinants of health.

## 1 THE HRRP AND OBSERVATION HOSPITALIZATIONS

The omission of observation hospitalizations from the HRRP “rehospitalization” metric is perhaps the most obvious shortcoming that has emerged.^6^, ^7^, ^8^, ^9^ Although counterintuitive, hospitalization is not synonymous with inpatient admission. Policymakers and clinicians less familiar with the hospital setting often confuse observation care as that which is limited to emergency department‐adjacent observation units. But observation stays commonly occur in hospital wards, indistinguishable from inpatient stays. One analysis determined that hospitalists provided the majority of observation care (59%), followed by traditional internal medicine and family medicine providers (21%), cardiology (10%), and emergency department providers (5%), with all other providers comprising the remainder (5%).^10^ To a patient, being hospitalized “under observation” means their stay is covered under Medicare Part B instead of Part A, and they lack post‐acute skilled nursing facility (SNF) coverage should they need it. To illustrate what this means to the HRRP, if a patient hospitalized for an HRRP target condition requires rehospitalization within 30 days, this will count as a readmission only if the index *and* re‐hospitalization are billed as an inpatient stay, but will be invisible under the HRRP if either hospitalization is billed as an outpatient observation stay. This is not just a theoretical problem: nearly one in five 30‐day rehospitalizations for HRRP target conditions are not counted under the HRRP due to observation stay as index, 30‐day rehospitalization, or both.^8^

The blurry line between observation and inpatient care has raised concerns about whether hospitals might strategically encourage physicians to categorize their patients as outpatient (observation) instead of inpatient (both of which are done by a physician order in the medical record) within 30 days of an index inpatient stay to improve their readmission rate. As part of the 21st Century Cures Act of 2016, Congress mandated the Medicare Payment Advisory Commission (MedPAC) study this question, with MedPAC concluding there was not “…a shifting of short‐stay admissions into observation stays to avoid readmission penalties” in their 2010–2016 analysis.^11^ Similarly, analyzing the years 2007–2015, Zuckerman and colleagues concluded that although observation care increased over time, physicians were not systematically classifying patients as outpatient observation instead of inpatient to improve their hospitals' readmission rate.^12^ Although it is possible that individual hospital behavior could be hidden in these big data analyses, both studies provided reassurance that this was not broadly occurring. Both studies included only index inpatient stays, followed by observation *or* inpatient stays within 30 days of the index inpatient stay.

Although key additions to the literature, many^6^, ^7^ called for studies with more comprehensive inclusion of observation stays in evaluations of HRRP performance—most specifically, including observation stays in *both* the index hospitalization and 30‐day rehospitalization counts. Recently, Sabbatini and colleagues^13^ evaluated this question and found that including observation stays in both the index hospitalization and 30‐day rehospitalization counts more than halved the perceived readmission improvements in targeted conditions.

## 2 NEW FINDINGS FROM WRIGHT AND COLLEAGUES

In this issue of Health Services Research, Wright and colleagues^14^ add key important findings to the observation question. They compare observation stay used as a proportion of total hospitalizations in target versus non‐target conditions using a 20% Medicare fee‐for‐service sample. Over the study years 2009–2016, observation stays increased from 2.2% to 4.0% in the 3 targeted conditions compared to 4.2%–5.8% of non‐target hospitalizations. For chronic obstructive pulmonary disease (COPD), observation stay use rose from 8.4% to 11.9% in the target group versus 5.3% to 6.6% in the control group. The authors found a small statistically significant but clinically insignificant (0.02% per month) greater increase in observation stays in the three‐condition target group as compared to control but no statistically significant difference in the COPD group. Because the growth in observation stay use was similar in target and non‐targeted groups, the authors conclude there was no deliberate and potentially inappropriate substitution of observation for inpatient stays to appear more favorable under the HRRP.

Although counterintuitive, this study adds to the body of literature confirming the problem of observation stays in the HRRP actually relates to the *appropriate* use of observation stays based on CMS rules and regulations. Here is why: the HRRP only counts inpatient stays, but the proportion of hospital stays now classified as outpatient observation has grown for reasons that likely include improved hospital efficiency resulting in acute‐care hospital stays shorter than two‐midnights designated as observation, and changes in visit status rules, such as hospital‐based procedures being removed from Medicare's “Inpatient‐Only List.” Thus, the problem of the HRRP is not misuse of observation but rather shifts in CMS policy that increases observation hospitalizations, further exposing the flaw in HRRP itself that fails to account for hospitalizations not billed as inpatient stays.

This distortion of the HRRP's rehospitalization metric has worsened over time as outpatient hospital care continues to grow. According to the MedPAC, from 2006 to 2016, outpatient services increased by 49.0% while inpatient discharges decreased by 21.8%.^15^ Although observation is only one type of outpatient service, observation hospital care is nonetheless a significant portion of hospital outpatient services. The impact of heterogeneous hospital visit types (inpatient, observation, or even emergency department visit) was illustrated by Wadhera and colleagues, who found that 30‐day hospital revisits actually increased for HRRP‐targeted conditions when both the emergency department and observation stays were counted alongside inpatient stays.^16^ The impact of increased observation stays is made even more complicated by variable observation use nationally, further distorting the HRRP's ability to measure quality.^17^

## 3 CAN THE HRRP BE IMPROVED?

Could the HRRP be improved if observation stays were included? The most obvious and significant implication would be on the current Medicare hospital reimbursement model. Under CMS' Outpatient and Inpatient Prospective Payment Systems (OPPS and IPPS), observation stays can have dramatically different hospital payments than inpatient stays, even if the primary diagnosis and actual services proved are similar.^18^ As a result, including observation stays in the rehospitalization metric would essentially be considering them inpatient stays for the purpose of penalties, but not payment—something that would likely face opposition from hospitals and providers. Similarly, because Medicare beneficiary post‐acute SNF Part A coverage requires a qualifying three‐night inpatient stay, patients and advocates would also have concerns about counting stays as inpatient for the purpose of a quality metric but not for the benefit of their SNF coverage.

Eliminating observation stays as a separate hospital visit status entirely—meaning all hospital stays would simply be considered hospital inpatient admissions—would be a significant improvement, but this degree of policy shift would have far‐reaching implications and is unlikely to happen. Additionally, solving the observation issue fixes only one of the many visit‐type or place of service issues under the HRRP. For example, ambulatory surgery center (ASC) use continues to grow, with one estimate projecting 25% growth between 2019 and 2029.^19^ These ASC encounters are also missing from readmission calculations because they do not serve as index stays, meaning the ASC has no financial consequences if a patient is subsequently admitted to a hospital as a complication of the procedure. Thus, even if including observation stays in the HHRP in some form is settled, as healthcare delivery continues to evolve, the HRRP may end up missing other patient categories and visit types.

Many other challenges remain. Although CMS “…encourages hospitals to improve communication and care coordination to better engage patients and caregivers in discharge plans and, in turn, reduce avoidable readmissions,”^2^ it is not clear this formula works, at least not for a full 30 days following discharge. This reality was illustrated by Graham and colleagues, who found that hospitals were the best location to influence early (0–7 days) readmissions, but less able to influence later (8–30 days) readmissions,^20^ when disease exacerbation unrelated to the hospitalization or other factors more directly related to under‐resourced areas or insufficient clinical follow up may play a larger role. Hospitals also typically do not receive any notification when patients are readmitted to a different facility, limiting the ability to analyze data and improve performance. Hospitals should be accountable for factors they can control, but the HRRP is too broad in scope and time to reflect these factors.

The growing proportion of Medicare Advantage (MA) enrollees also changes the readmission dynamic. When the HRRP began in 2012, 27% of Medicare beneficiaries were enrolled in an MA plan. In 2022, that number was 48%.^21^ While FFS Medicare will pay for a new readmission diagnosis‐related group (DRG), most MA plans will not. With MA enrollment projected to soon exceed 50%, this is another reason the readmission landscape is significantly different for a hospital or health system than when the HRRP began.^22^

Most importantly, the HRRP has failed to adequately account for social determinants of health. Living in a disadvantaged neighborhood predicts readmissions to a degree similar to having a chronic medical condition, like peripheral vascular disease.^23^ The CMS acknowledges that under‐resourced populations experience increased readmissions, and has issued related guidance and recommendations in their “Guide to Reducing Disparities in Readmissions”.^24^ Yet these recommendations require resources—resources that ironically may actually be taken away from hospitals serving under‐resourced populations under the HRRP. In the words of Figueroa and colleagues “At best, the evidence to date suggests that the HRRP has had no meaningful effect on the rate at which patients return to the hospital within 30 days of discharge. At worst, the HRRP has unfairly penalized hospitals caring for the most vulnerable populations in our country and potentially resulted in patient harm.”^25^ Cram and colleagues concur, stating that “Persistent focus on readmissions…has had minimal demonstrable benefit. Moreover, the HRRP has distracted clinicians and health system leaders from other crucial quality concerns,”…and has not sufficiently addressed the “…critical contribution of disadvantage and adverse social determinants of health in driving hospital readmissions at both the individual and hospital level.”.^26^

## 4 CONCLUSION AND POLICY RECOMMENDATIONS

The HRRP is begging for reform, yet what is the solution? In February 2023, the CMS signaled its intent to include all‐cause readmissions in its Universal Foundation of aligned quality measures.^27^ Thus, eliminating the HRRP is an unlikely policy option, and efforts should be directed at improving the measurement to address program concerns. From a health disparities perspective, CMS could track readmissions but eliminate penalties for hospitals that invest resources to address elements identified in their Guide to Reducing Disparities in Readmissions.^24^ Indeed, the HRRP penalties themselves have failed to distinguish quality over the past decade, as the majority (93%) of hospitals have been penalized since the program started, with almost half (47%) penalized in 2022 alone.^28^, ^29^ Rather than penalize hospitals caring for the most under‐resourced beneficiaries under the HRRP, CMS could prioritize their Guide recommendations with clear, actionable steps to help hospitals know where best to use resources and how to improve their community‐based partnerships. Hospitals spend an average of $300–600 per patient to reduce 30‐day readmissions^30^; re‐investing these dollars and those currently used to pay HRRP penalties in these community‐based partnerships and local efforts in the ways most beneficial to individuals could be a better and more impactful use of the Medicare Trust Fund. CMS should also continue to explore ways to provide additional resources to hospitals serving the most under‐resourced beneficiaries through innovative models like the Accountable Care Organization Realizing Equity, Access, and Community Health (ACO REACH), where per beneficiary per month benchmark adjustments are made.^31^ CMS might also enhance their current inpatient payment system, which offers additional monies to hospitals taking care of under‐resourced populations through its Disproportionate Share Hospital adjustment. Ultimately, moving away from a punitive HRRP system might alleviate some hospital and beneficiary concerns about an “all‐rehospitalization” metric inclusive of observation stays, ASC visits, and perhaps emergency department visits.

“If you can't measure it, you can't improve it”—applies to the performance of the HRRP itself. The HRRP has been measured for over a decade and the program is in dire need of a comprehensive overhaul. Legislators and regulators must engage in efforts to improve the HRRP on the policy level so it can truly deliver on its stated goal of improving hospital and healthcare quality.

## FUNDING INFORMATION

Dr. Powell receives support from the National Institute on Aging (R21AG079277) unrelated to this article. Dr. Sheehy receives support from the National Institute on Aging (3RO1AG070883‐02) unrelated to this article. This material is the result of work supported by the resources and the use of facilities at the University of Wisconsin School of Medicine and Public Health Center for Health Disparities Research. The content is solely the responsibility of the authors and does not necessarily represent the official views of the National Institutes of Health.
